# Cocaine amphetamine-regulated transcription peptide inhibits apoptosis in oxygen–glucose deprived neural stem cells

**DOI:** 10.3389/fnins.2024.1424719

**Published:** 2024-08-20

**Authors:** Lu Zhang, Shan Chen, Renfang Zou, Xin Shu, Jingxuan Zhang, Xuan He, Moxi Su, Luna Wang, Bin Wang, Dujuan Sha

**Affiliations:** ^1^Department of General Practice, Nanjing Drum Tower Hospital Clinical College of Nanjing University of Chinese Medicine, Nanjing, Jiangsu, China; ^2^Department of General Practice, Nanjing Drum Tower Hospital, Affiliated Hospital of Medical School, Nanjing University, Nanjing, Jiangsu, China; ^3^The State Key Laboratory of Pharmaceutical Biotechnology, Nanjing University, Nanjing, Jiangsu, China; ^4^Clinical Stem Cell Center, Nanjing Drum Tower Hospital, Affiliated Hospital of Medical School, Nanjing University, Nanjing, Jiangsu, China; ^5^Department of General Practice, Nanjing Drum Tower Hospital Clinical College of Nanjing Medical University, Nanjing, Jiangsu, China

**Keywords:** cocaine amphetamine-regulated transcription peptide, neural stem cells, apoptosis, oxygen glucose deprivation, CAMP-response element binding protein

## Abstract

**Background:**

Apoptosis has been recognized as a critical pathophysiological process during cerebral ischemia. The neuroprotective effect of CART on ischemic brain injury is determined. However, there is little research on the protective effect of CART on neural stem cells (NSCs).

**Methods:**

Primary cultured rat NSCs were utilized as the research subject. *In vitro* oxygen glucose deprivation (OGD) treatment was employed, and NSCs were extracted from SD pregnant rats following previous experimental protocols and identified through cell immunofluorescence staining. The appropriate concentration of CART affecting OGD NSCs was initially screened using Cell Counting Kit-8 (CCK-8) and Lactate Dehydrogenase (LDH) assays. EdU staining and Western blotting (WB) techniques were employed to assess the impact of the suitable CART concentration on the proliferation and apoptosis of OGD NSCs. Finally, Western blot analysis was conducted to investigate the cAMP-response element binding protein (CREB) pathway and expression levels of related proteins after KG-501 treatment in order to elucidate the mechanism underlying apoptosis and proliferation regulation in OGD NSCs.

**Results:**

CCK-8 and LDH assays indicated that a concentration of 0.8 nM CART may be the optimal concentration for modulating the proliferation of OGD NSCs. Subsequently, cellular immunofluorescence and EdU detection experiments further confirmed the findings obtained from CCK-8 analysis. Western blot analysis of apoptosis-related protein expression also demonstrated that an appropriate concentration of CART could suppress the apoptosis of OGD NSCs. Finally, Western blotting was conducted to examine the CREB pathway and related protein expression after treatment with KG-501, revealing that an appropriate concentration of CART regulated both apoptosis and proliferation in OGD NSCs through CREB signaling.

**Conclusion:**

The concentration of CART at 0.8 nM may be deemed appropriate for inhibiting apoptosis and promoting proliferation in OGD NSCs *in vitro*. The mechanism maybe through activating the CREB pathway.

## 1 Introduction

Globally, the prevalence of ischemic stroke is high, making it a prominent cerebrovascular disease and a leading cause of death and disability worldwide. Ischemic stroke typically occurs due to focal cerebral hypoperfusion caused by embolic or atherosclerotic factors. Although there is temporary interruption in blood supply to the brain tissue, spontaneous restoration of blood flow may lead to permanent tissue damage while providing some relief (Powers, 2020). The ischemia may progress to an irreversible cerebral infarction if blood flow to the blocked artery is not restored in a timely manner (Pandian et al., 2018). The occurrence of ischemic strokes is more prevalent among adults, leading to a wide range of neurological deficits that significantly impact their quality of life and functional abilities. Moreover, this places a substantial economic burden on both the affected individuals’ families and society as a whole (Campbell and Khatri, 2020). There will be a global increase in the number and incidence of ischemic stroke in both men and women, but the incidence will still be higher in women than in men. A previous study also demonstrated that women have a higher lifetime risk of stroke than men in younger age groups, that the incidence of ischemic stroke is higher in men than in women, and that this pattern may be reversed after middle age due to the onset of menopause and lower estrogen levels in women. In addition, women are less likely to be diagnosed and treated for stroke than men, perhaps because women are more likely to present with atypical stroke symptoms and are less likely to know about them, or perhaps because they receive less social support than men. These atypical symptoms are rarely considered warning signs of stroke, resulting in a missed stroke diagnosis (Pu et al., 2023). Although the incidence of ischemic stroke is substantial, the available treatment options remain severely limited (Zerna et al., 2018). Currently, the common clinical treatments for ischemic stroke commonly involve the administration of thrombolytic drugs, such as tissue-type fibrinogen activators, and mechanical removal of blood clots through surgical embolization (Powers, 2020). Recombinant tissue plasminogen activator (r-tPA) is the only effective Class 1A drug approved by the U.S. Food and Drug Administration for the treatment of acute ischemic stroke, but it has a very narrow therapeutic window (generally within 4.5 h) and is associated with treatment-related complications (cerebral hemorrhage) and contraindications (Ning et al., 2010). Therefore, the utilization and promotion of intravenous thrombolytics are significantly restricted. In patients with contraindications to intravenous thrombolysis and a heightened risk of bleeding from thrombolysis, endovascular interventions (such as endovascular thrombectomy) may serve as an adjunct or alternative treatment for early reocclusion of major vessels (Yoshimura et al., 2022). The addition of this adjuvant treatment can potentially extend the therapeutic window up to 12 h, while also potentially enhancing functional independence and revascularization rates in comparison to r-tPA treatment alone (Huo et al., 2023). The promotion of endovascular interventions, however, faces challenges due to the high equipment requirements and the significant impact of treatment duration on efficacy. Additionally, less than 10% of patients meet the criteria for endovascular intervention (Mazighi et al., 2009).

In the last few decades, the identification of post-stroke neurogenesis has paved the way for a novel therapeutic approach in managing ischemic stroke (Flax et al., 1998). The process of neurogenesis involves the generation of new neurons to replace damaged ones in the adult brain following injury (Ottoboni et al., 2020). The central nervous system harbors a population of cells known as NSCs, which possess the remarkable ability to self-renew and exhibit multipotency, enabling them to differentiate into various lineages including neurons, astrocytes, and oligodendrocytes (Gonçalves et al., 2016). Newly generated neurons have the capacity to integrate into the impaired neural network, newly formed astrocytes can exert nourishing, supportive, and protective functions in the restoration of the neural network, while newborn oligodendrocytes are capable of repairing the damaged myelin sheath (Hamblin and Lee, 2021). When the brain sustains damage, such as traumatic brain injury (TBI) or ischemic stroke, it triggers the activation of endogenous NSCs (eNSCs), leading to their proliferation. Subsequently, neuroblasts migrate towards the site of injury and eventually differentiate into fully functional mature neurons that seamlessly integrate into the damaged neural network (Rahman et al., 2021). Although some neuronal regeneration occurs after stroke, the number of new neurons produced is extremely limited to achieve complete repair (Cuartero et al., 2021). The therapeutic modalities for treating ischemic stroke using NSCs include implanting exogenous NSCs and using endogenous NSCs to induce neurogenesis in the semi-dark zone of the infarcted area (Zhao et al., 2022). Compared to endogenous NSCs, exogenous implantation presents significant drawbacks in terms of immune rejection, tumorigenicity, and ethical considerations (Zhang et al., 2019). Therefore, the development of novel therapeutic strategies aimed at enhancing neural regeneration may serve as a pivotal approach to unraveling the mechanisms underlying neural recovery following cerebral ischemia.

Cocaine amphetamine-regulated transcription peptide is a neuropeptide, which is expressed in neural and endocrine tissues (Zhang et al., 2012). In the brain, the CART peptide is primarily localized in the hypothalamus, midbrain, and hippocampus within the brain, as well as in the ventral striatum (Ahmadian-Moghadam et al., 2018). CART exhibits strong correlations with a range of cerebral functions, encompassing drug addiction, appetite regulation, and neuroprotection (Ahmadian-Moghadam et al., 2018). In recent years, numerous research studies have demonstrated the neuroprotective effects of CART in ischemic brain injury. Specifically, during middle cerebral artery occlusion (MCAO), it has been observed to effectively reduce the volume of cerebral infarction. Additionally, when applied *in vitro* to cortical neurons, CART has shown a significant decrease in neuronal cell death induced by oxygen–glucose deprivation. The knockdown of CART expression by shRNA resulted in an increase in ischemic cerebral infarct volume and neuronal cell death following oxygen–glucose deprivation in mice (Xu et al., 2006; Sha et al., 2013). CART is associated with the modulation of dopamine in ischemic stroke and mitigates brain damage following ischemic stroke by attenuating inflammatory activation (Lin et al., 2018). The results of our preliminary studies indicate that CART exerts neuroprotective effects on oxygen–glucose-deprived neurons through the inhibition of oxidative stress and enhancement of mitochondrial complex II activity (Sha et al., 2014). CART protects synaptic plasticity in neurons after ischemic cerebral injury (Zhang et al., 2020). The latest research findings have demonstrated that the intranasal administration of CART has the potential to induce neuroregeneration in rats with stroke (Luo et al., 2013). To date, there have been no relevant reports elucidating the mechanism by which CART induces nerve regeneration following ischemic stroke, nor have there been any studies investigating the optimal concentration of OGD NSCs treated with CART *in vitro*.

The present study initially addresses the optimal concentration of CART in the treatment of OGD NSCs, subsequently examines the impact of an appropriate dose of CART on apoptosis and proliferation in OGD NSCs, and ultimately investigates the specific mechanism underlying the effects of an appropriate concentration of CART on apoptosis and proliferation in OGD NSCs. Consequently, this study aims to elucidate how CART induces nerve regeneration following ischemic stroke along with its intrinsic mechanisms, thereby offering a novel theoretical foundation for the development of CART as a therapeutic agent for ischemic stroke.

## 2 Materials and methods

### 2.1 Materials

CART_55-102_ was obtained from Phoenix Pharmaceuticals (Belmont, California, United States). Antibodies against Nestin, Sox, Synaptophysin CREB and phosphor-CREB (phosphor S133) were purchased from Abcam (Cambridge, MA, United States). Antibodies against Tubulin was purchased from Beyotime Biotechnology (Shanghai, China). KG501 ([3-[(4-chlorophenyl) carbamoyl]-naphthalen-2-yl] dihydrogen phosphate) was from Sigma-Aldrich (St. Louis, MO, United States). Antibodies against Bcl-2, Bax were purchased from Bioworld Biotechnology (Minneapolis, MN, United States).

### 2.2 Primary cell culture

The E13-15 SD pregnant mice was anesthetized with 4% chloral hydrate, and the fetal mice were exposed. The brain tissue and hippocampus were separated, cut into small pieces and placed in a 37°C incubator with Accutase enzyme to digestion for 20 min. The NSCs were cultured into the DMEM/F12 complete media supplemented with 2% B27, 1% penicillin–streptomycin, 20 ng/mL EGF and BFGF for re-suspension and mixing, and then seeded into a T25 culture flask after filtration at 37°C, 5% CO2. Cell media was added in time and cells were passed when the diameter of nerve ball reaches 100 μm.

### 2.3 Oxygen glucose deprivation and reperfusion

The NSCs were digested into single cells and seeded into plates. The complete media containing 10% FBS was added for adherent culture. After the cells were attached to the wall, the old media was removed and glucose-free media was added. The plate was incubated in a hypoxia chamber containing 5% CO2 and 95% N2 for half an hour at 37°C. Then the glucose-free medium of cells were replaced with original medium and incubated for another 18 h for future experiments.

### 2.4 Cell viability

NSCs were seeded into 96-well plates (30,000 cells per well). After OGD/R treatment, 100 μL of complete culture media containing 10% CCK-8 solution was added to each well and incubated for 4 h at 37° C. Absorbance was measured at 450 nm with an microplate reader.

### 2.5 LDH

NSCs were seeded into 96-well plates (30,000 cells per well). After the OGD/R treatment, adhere to the instructions provided by the LDH kit. Firstly, prepare the LDH reaction mixture solution required for the experiment in advance. Then, aspirate the complete medium from each well of the 96-well plate and add 100 μL of LDH reaction mixture solution to each well. Subsequently, place the 96-well plate in a constant temperature incubator at 37°C and 5% CO_2_ for a light-free incubation period of 30 min. Following this dark incubation step, add 50 μL of termination solution to each well of the 96-well plate and gently shake it for approximately 10 s to ensure complete reaction. Finally, measure the absorbance value at wavelengths of 490/492 nm for each well.

### 2.6 Immunofluorescence

NSCs were seeded into a pre-coated poly-L-Lysine 24-well plate (100,000 cells per well). After adherent culture and OGD/R treatment, cells were fixed with 4% formaldehyde at room temp for 30 min and then rinsed with PBS. 0.25% Triton X-100 was added and left to incubate for 20 min. NSCs were blocked with 5% BSA for 1 h. Approximately 500 μL of diluted primary antibodies (Nestin, Mouse, Abcam#ab6142, 1/5,000), (Sox, Rabbit, Abcam#ab97959, 1/1,000) was added to each well after buffer removal and incubated overnight at 4°C. The next day primary antibodies were removed and the wells rinsed 3 × 5 min with sterile PBS. Secondary antibodies (Alexa#11031 Nestin with Goat Anti-Mouse 568 red), (Alexa#11034 Sox with Goat Anti-Rabbit 488 green) were added to the 24-well plate and incubated at room temp for 1 h. The coverslips with cells were washed with PBS and placed on the slides containing the Mounting Media with DAPI, Aqueous and Fluoroshield (Abcam#ab104139) to dry. Imaging was performed through Confocal inverted microscope at 10x and 40x magnification.

### 2.7 Western blotting

The cell pellets were granulated by adding RIPA (Beyotime Biotechnology, China), and centrifuged at 4°C for 20 min to collect the supernatant (protein). Protein concentration was quantified by BCA Kit (Vazyme, China). 10 μg proteins were heated at 95°C for 10 min before being loaded into a Gel tank (12% gradient gel), and run for 70 volts for 10 min followed by 120 volts for 75 min. The protein gel was removed and placed on the PVDF membrane for transfer. The membrane was blocked in blocking buffer (5% skim milk + PBST) for 1 h. After the blocking, primary antibodies (1/1000 of Bcl-2, Bax, Tubulin, T-CREB and P-CREB) were added and the blot was incubated overnight at 4°C. The next day, the blot was rinsed with 3× 5 min washes in PBST and secondary antibody was added. The blot was then incubated for 1 h at room temp. The membrane washed with PBST and sufficiently covered with ECL Chemiluminescence Kit (Vazyme, China), and finally placed into the Tanon Imaging System.

### 2.8 EdU staining

The proliferation of NSCs was detected by the Cell-Light EdU Apollo *In Vitro* Kit (Ribobio, Guangzhou, China) according to the instructions. Briefly, the NSCs were incubated with a complete culture medium containing EdU for 24 h and fixed at room temperature for 30 min. Glycine was added to each well to neutralize the excess aldehyde group, then 0.5% Triton-X was added and left to incubate for 10 min. The dye reaction solution was added and incubated for half an hour in dark, and then washed with 0.5%Triton-X for 3 ×10 minutes. Then the coverslips with cells were placed on the slides containing the Mounting Media with DAPI, Aqueous and Fluoroshield (Abcam # ab104139) to dry. Imaging was performed through Confocol inverted microscope at 10x and 40x magnification.

### 2.9 Statistics analysis

The experimental data were represented as mean ± SEM. Statistical analysis among groups was done by one-way ANOVA followed by Dunnett’s multiple comparison test. A value of *p* < 0.05 was considered statistically difference.

## 3 Results

### 3.1 The concentrations of CART required for the protection of OGD NSCs *in vitro* were determined using CCK-8 and LDH assays

**Figure 1:**
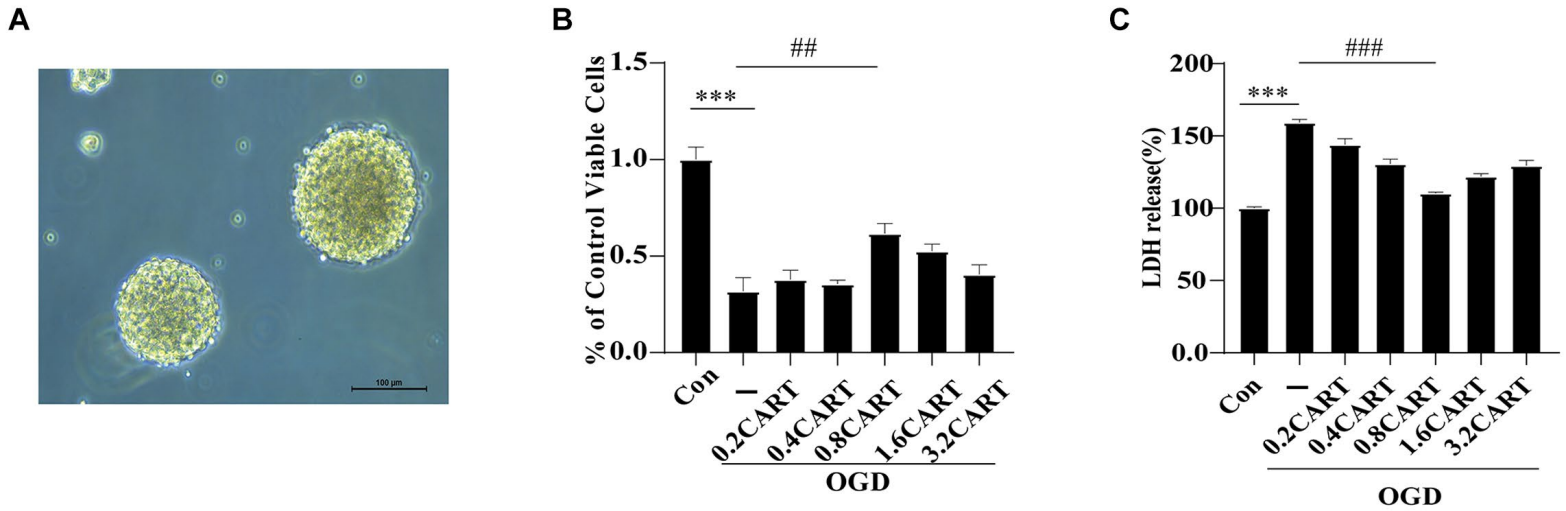
CART protects NSCs from oxygen glucose deprivation. **(A)** The morphological mapping of NSCs under a microscope. **(B)** NSCs were treated with CART55-102 during OGD, cell viability of NSCs was measured using the CCK-8 assay. Date is expressed as mean ± SEM. The experiment was performed in triplicate. **(C)** Cytotoxicity of NSCs was measured using the LDH assay. Date is expressed as mean ± SEM. The experiment was performed in triplicate. ^***^p < 0.001, ^##^*p* < 0.01, ^###^*p* < 0.001.

To validate the optimal concentration of CART_55-102_ for its protective effect on NSCs subjected to OGD *in vitro* (Figure 1A), we cultured primary NSCs under OGD conditions and treated them with varying concentrations of CART_55-102_:0.2 nM, 0.4 nM (based on our previous study data), 0.8 nM, 1.6 nM, and 3.2 nM.The CCK-8 assay was employed to evaluate the activity of NSCs in each group, while LDH assay was utilized to determine the level of LDH released in each group, thereby assessing the cytotoxicity and damage extent of NSCs across groups. As anticipated, primary NSCs exhibited significantly reduced activity levels under OGD exposure compared to normal controls (^***^p < 0.001). However, CART_55-102_ treatment markedly enhanced primary NSCs activity and mitigated cytotoxic effects on these cells relative to the OGD group. Notably, among CART_55-102_-treated groups, the 0.8 nM concentration demonstrated the most pronounced effect. The experimental results of CCK-8 demonstrated that 0.8 nMCART_55-102_ significantly augmented cellular viability and improved the survival rate of OGD NSCs (Figure 1B). LDH findings revealed that 0.8 nMCART_55-102_ markedly attenuated cytotoxicity in OGD NSCs and mitigated the extent of injury (Figure 1C). Both sets of results confirmed that an optimal concentration of CART for *in vitro* protection against OGD-induced damage in NSCs was 0.8 nM.

### 3.2 The optimal concentration of CART enhanced the *in vitro* neuroprotection and proliferation of OGD NSCs

**Figure 2:**
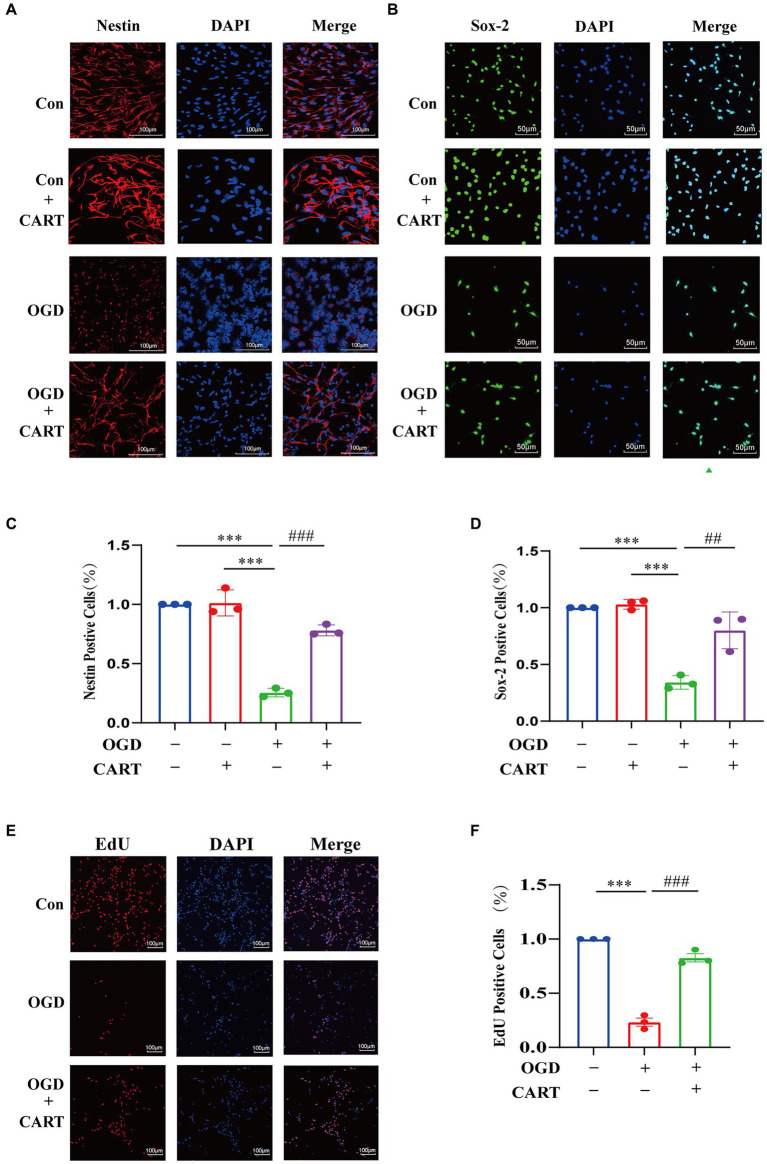
CART treatment protects NSCs and enhances proliferation of NSCs in oxygen and glucose deprivation. **(A)** CART55-102 (0.8 nM) treatment inhibits loss of neural stem cell structures (Nestin, Red) and increases Nestin (Red) expression in cultured NSCs. Scale bars = 100 μm. **(B)** CART55-102 (0.8 nM) treatment enhances Sox-2 (Green) expression in cultured NSCs. Scale bars = 50 μm. **(C)** Quantitative analysis of Nestin fluorescence results using ImageJ software, date was presented as mean ± SEM, ***p < 0.001, ###*p* < 0.001. **(D)** Quantitative analysis of. Sox-2 fluorescence results using ImageJ software, date was presented as mean ± SEM, ***p < 0.001, ##*p* < 0.01. **(E)** EdU staining detects proliferation of oxygen and glucose deprived NSCs. Scale bars =100 μm. **(F)** Quantitative analysis of fluorescence results using ImageJ software, date was presented as mean ± SEM, ***p < 0.001, ###*p* < 0.001. Nestin: Neuroepithelial stem cell protein; Sox-2: Sex determining region Y box 2.

To investigate the effect of CART on the structure and survival of OGD NSCs, we performed primary NSCs cultures. CART_55-102_ (0.8 nM) was added after OGD treatment. Immunofluorescence staining was performed using nestin-specific antibody and Sox-2-specific antibody. The findings revealed that the cell structure of NSCs was damaged, cells growth was significantly inhibited and the number of viable cells was decreased. CART treatment reverses the inhibitory effects of OGD on the structural damage and growth of NSCs (Figures 2A–C) and increases the number of surviving cells (Figures 2B–D). In order to further verify the proliferative effects of CART on OGD NSCs, we used the EdU staining. Staining results revealed that the cell number of OGD NSCs was significantly decreased and the proliferative viability of the cells was decreased. CART treatment increased the cell number of OGD NSCs and significantly increased the proliferative viability of the cells (Figure 2E), suggesting that CART has a proliferative enhancing effect on OGD NSCs (Figure 2F).

### 3.3 CART effectively inhibits apoptosis in OGD NSCs

**Figure 3:**
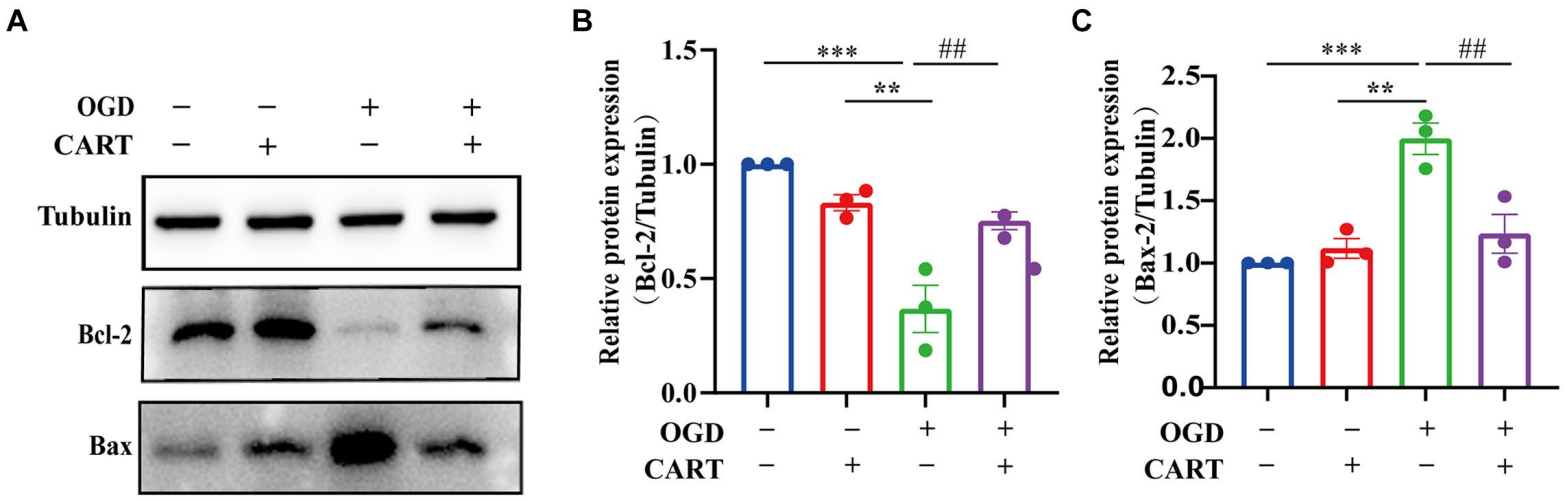
CART inhibits the level of apoptosis in NSCs of the OGD. **(A)** The protein levels of Bcl-2 and Bax were measured by Western bolt in different groups. **(B)** Quantitative analysis of Bcl-2 expression. The blot shown here is a representative 3 independent experiments. The blot images were cropped for comparison. All values are expressed as mean ± SEM of the band intensity of Bcl-2 normalized to Tubulin from 3 independent experiment. **(C)** Quantitative analysis of Bax expression. Results were presented as mean ± SEM.**p < 0.01, ***p < 0.001, ##*p* < 0.01, ###*p* < 0.001. Bcl-2: B-cell lymphoma-2; Bax: BCL2-Associated X protein.

Bcl-2 and Bax are considered to be the most important genes involved in the process of apoptosis. Bcl-2 family proteins play an important role in the regulation of apoptosis by forming dimers with Bax as well as dimerizing themselves. When Bcl-2 protein is inhibited, it forms fewer dimers with Bax, leading to apoptosis. When Bcl-2 protein is overexpressed, it forms more heterodimers with Bax, and apoptosis is inhibited. Bax is an apoptosis-related gene that forms heterodimers with bcl-2, and its overexpression inhibits the function of bcl-2 and promotes apoptosis. The next research investigated whether the neuroprotective effect of CART_55-102_ (0.8 nM) on OGD was related to the level of apoptosis. We evaluated the protein levels of Bcl-2 and Bax. The results revealed a decrease in Bcl-2 expression and an increase in Bax expression after OGD, whereas an increase in Bcl-2 expression and a decrease in Bax expression were observed after treatment with CART_55-102_ (0.8 nM) (Figure 3A) Significant differences in Bcl-2 and Bax expression were observed between the CART group and the OGD group and the control group (*p* < 0.01, Figures 3B,C).

### 3.4 CART upregulated p-CREB levels in OGD NSCs

**Figure 4:**
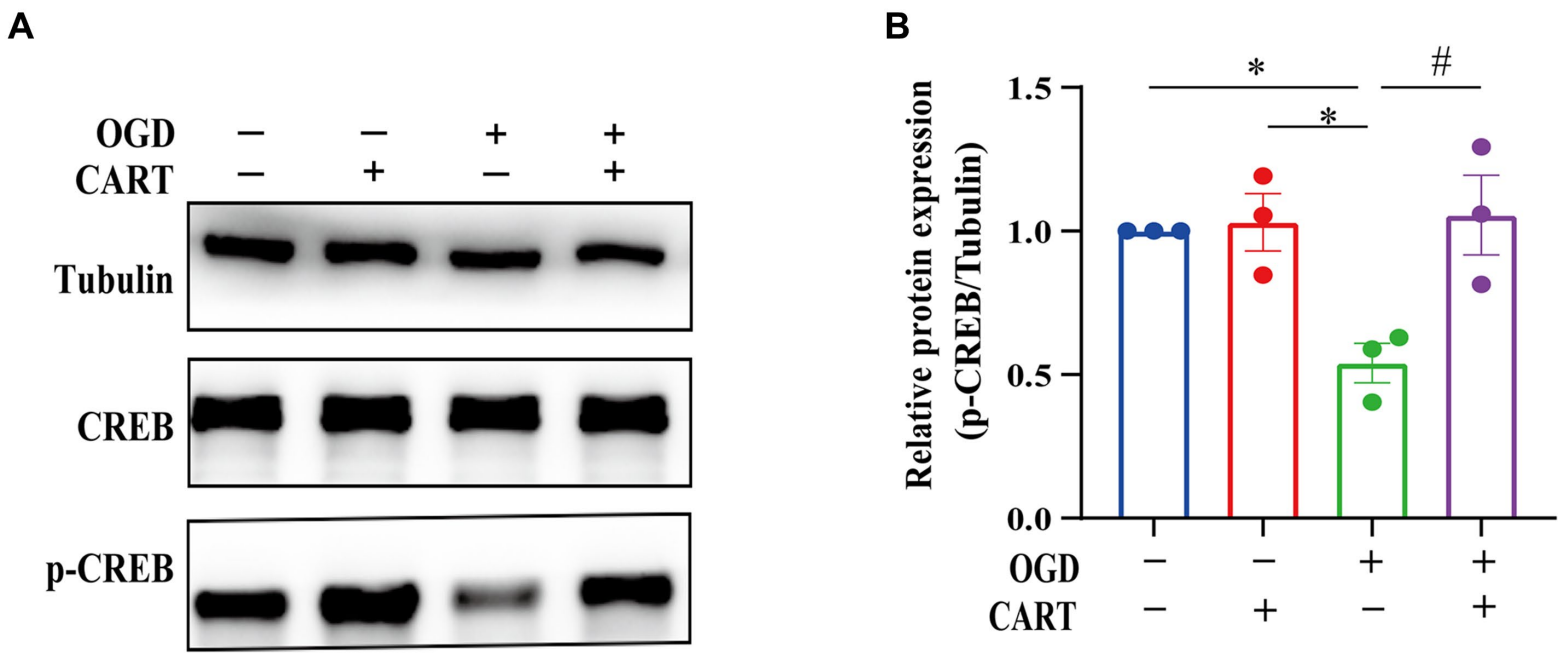
CART upregulates p-CREB levels in NSCs deprived of OGD. **(A)** The protein levels of CREB and p-CREB were measured by Western blot. **(B)** Quantitative p-CREB expression analysis. The blot shown is representative of 3 independent experiments. All values are expressed as mean ± SEM of band intensity of p-CREB normalized to Tubulin from 3 independent experiments. *p < 0.05, #*p* < 0.05. p-CREB: phospho-cAMP response element binding protein.

Activated cAMP response element binding protein (CREB) is a crucial regulator of nerve growth and development. The results of the next investigation revealed a significant decrease in p-CREB expression in the OGD group. p-CREB expression was significantly different between the OGD group and the normal group (*p* < 0.05), whereas p-CREB expression was upregulated in the CART group after treatment with CART_55-102_ (0.8 nM, Figure 4A). p-CREB expression was significantly different between the CART group and the OGD group and the control group (*p* < 0.01). There was no change in total CREB expression (Figure 4B). These results demonstrate that CART_55-102_ reversed the decrease in p-CREB expression after OGD.

### 3.5 KG-501 inhibited cell proliferation and enhanced apoptosis of OGD NSCs

**Figure 5:**
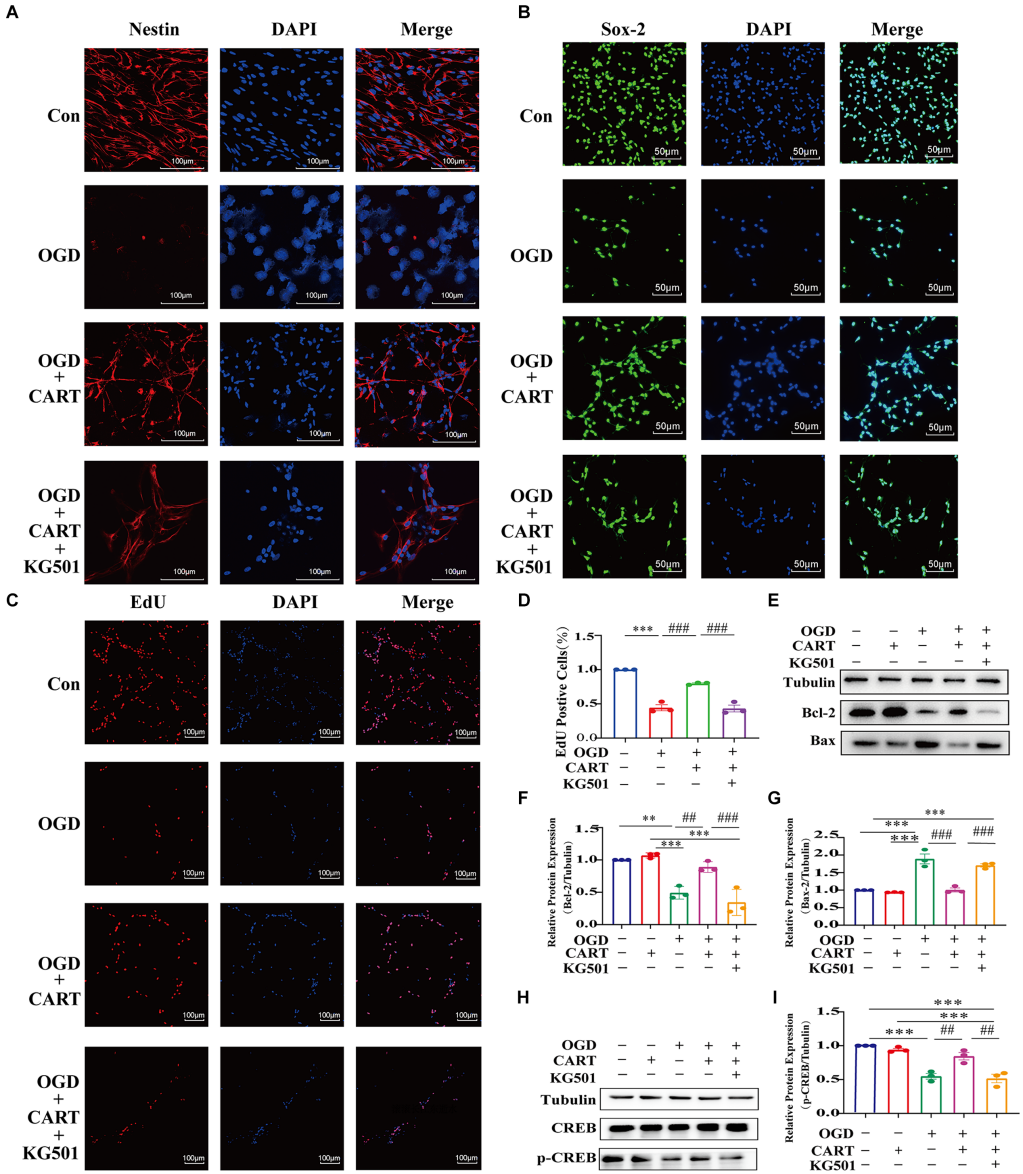
The effect of KG501 (a specific inhibitor p-CREB) on proliferation and apoptosis levels in OGD NSCs. **(A)** CART55-102(0.8 nM) treatment protects against loss of the structure of NSCs (Nestin, red) and enhances Nestin (Red) expression and KG501 inhibits the protection of structure in cultured oxygen and glucose deprived NSCs. Scale bar = 100 pm. **(B)** CART55-102(0.8 nM) treatment enhances Sox-2(Green) expression and KG501 inhibits the protection of neural stem cells in cultured oxygen and glucose deprived. Scale bar = 50 μm **(C)** CART55-102(0.8 nM) treatment increases the proliferation of oxygen and glucose deprived neural stem cells and KG-501 inhibits the proliferation of NSCs in cultured oxygen and glucose deprived. Scale bar = 100um. **(D)** Quantitative analysis of fluorescence results using ImageJ software. Data was presented as mean ± SEM, ***p < 0.001, ###*p* < 0.001. **(E)** The protein levels Bcl-2 and Bax were measured by Western blot in different groups. **(F)** Quantitative analysis of Bcl-2 expression. Blot images were cropped for comparison. All values are expressed as mean ± SEM of the band intensity of Bcl-2 normalized to Tubulin from 3 independent experiments. Compared with the OGD, after treatment CART55-102 (0.8 nM), the levels of Bcl-2 significantly increased. But the levels of Bcl-2 were significantly decreased after pre-treatment with KG501 in the CART treatment group. **p < 0.001, ***p < 0.01, ##*p* < 0.01, ###*p* < 0.001. **(G)** Quantitative analysis of Bax expression. Blot images were cropped for comparison. All values are expressed as meant ± SEM of the band intensity of Bax normalized to Tubulin from 3 independent experiments. Compare with OGD, after treatment CART55-102(0.8 nM), the levels of Bax significantly decreased. But the levels of Bax were significantly increased after pre-treatment with KG501 in the CART treatment group. ***p < 0.001, ###*p* < 0.001. **(H)** The protein levels of p-CREB were measured by Western blot in different groups. **(I)** Quantitative analysis p-CREB expression. Blot images were cropped for comparation. All values are expressed as mean ± SEM of the band intensity of p-CREB normalized to 3 independent experiments. Compare with the OGD, after treatment CART55-102(0.8 nM), the levels of p-CREB significantly increased. But the levels of p-CREB were significantly decreased after pre-treatment with KG501 in the CART treatment group. ***p < 0.001, ##*p* < 0.01.

KG-501 is a specific CREB inhibitor. Immunofluorescence staining with nestin-specific and Sox-2-specific antibodies demonstrated that NSCs growth was significantly inhibited and cell viability was significantly decreased in the KG501-treated CART group in comparison to the CART group (Figures 5A,B). Similarly, EdU immunofluorescence staining showed a decline in cell number and a significant decrease in the level of cell proliferation of NSCs in the KG-501-treated CART group when compared to the CART group (*p* < 0.001, Figures 5B,C). In addition, We also observed that Bcl-2 and p-CREB protein expression was significantly downregulated and Bax protein expression was significantly upregulated in the KG-501-treated CART group compared to the CART group (Figures 5E–I). This result indicates that CART may achieve neuroprotection against ischemic cerebral injury by activating CREB to enhance the proliferation of OGD NSCs and inhibition of apoptosis of OGD NSCs.

## 4 Discussion

CART is widely distributed and abundantly expressed, playing a crucial role in the regulation of various physiological functions in both humans and animals since its discovery in 1995 (Douglass et al., 1995). The CART peptide encompasses a plethora of fragments, including CART_10-89_, CART_55-102_, CART_62-102_, CART_85-102_, and CART_89-103_. Among these fragments, the expression of CART_55-102_ is widely observed in brain tissues (Dominguez, 2006). The highest level of expression is found in the hypothalamus. Hippocampus, ventral striatum and cerebral cortex are also expressed (Ahmadian-Moghadam et al., 2018). CART is implicated in a diverse array of physiological functions, encompassing regulation of food intake and body weight, processing of sensory information, endocrine control and stress modulation, maintenance of energy balance and susceptibility to drug addiction, facilitation of reward and reinforcement mechanisms, regulation of anxiety responses, as well as promotion of neuronal growth (Mao et al., 2012; Salinas et al., 2014; Choudhary et al., 2018). The study has found that estradiol has a neuroprotective effect on ischemic neurons through upregulation of CART expression (Xu et al., 2006). The subsequent study demonstrated that CART effectively attenuated oxidative stress in OGD neurons by enhancing the activity of mitochondrial complexes II (Sha et al., 2014). CART plays important role in neuronal synaptic plasticity after ischemic cerebral injury (Zhang et al., 2020). Intranasal administration of CART 3 days after MCAO increased brain CART levels, up-regulated BDNF expression, enhanced NSCs survival, proliferation and migration, improved neurological function, and promoted nerve regeneration in stroke animals (Luo et al., 2013). However, there is currently a lack of reported information regarding the effects of CART on OGD NSCs *in vitro* and the optimal concentration for CART treatment. In this study, we treated OGD NSCs with varying concentrations of CART and divided them into different groups. Cell activity and cytotoxicity were assessed in each group using CCK-8 and LDH experiments. The results demonstrated a significant decrease in cell activity and an increase in cytotoxicity following OGD treatment. Nevertheless, treatment with different concentrations of CART led to a substantial increase in cell activity and a notable reduction in cytotoxicity across all groups. Notably, 0.8 nMCART treatment exhibited the most pronounced effect among the various concentration treatments. Therefore, through our CCK-8 and LDH experiments, we successfully identified the appropriate exogenous CART concentration for treating OGD NSCs *in vitro* while fully confirming its protective effect against OGD-induced damage to these cells.

The high mortality and disability rates associated with ischemic stroke make it a significant health threat (Campbell and Khatri, 2020; Powers, 2020). The primary clinical treatments for ischemic stroke include acute intravenous thrombolysis and endovascular intervention, both of which have a limited therapeutic window and can potentially result in complications (Pandian et al., 2018; Zerna et al., 2018). There is currently no effective treatment available for ischemic stroke. Ischemic stroke triggers a range of detrimental pathophysiological changes in and around the infarct area, including disruption of the blood–brain barrier, formation of cerebral edema, infiltration of local immune inflammatory cells, and production of reactive oxygen species. These processes can result in neuronal injury and necrosis as well as disruption of the axonal network (Kuroiwa et al., 1985; Yemisci et al., 2009; Iadecola and Anrather, 2011). The pathophysiological mechanisms underlying nerve injury in ischemic stroke are intricate, and nerve regeneration plays a pivotal role in the process of ischemic injury and repair, closely intertwined with functional recovery following an ischemic stroke. NSCs, a specific type of stem cells found within the nervous system, assume a crucial function in the restoration of neurological disorders. NSCs possess several advantageous characteristics including high self-renewal capacity, potential for multidirectional differentiation, excellent tissue compatibility, and low immunogenicity (Yandava et al., 1999). The findings from previous studies have demonstrated the significant efficacy of NSCs in the treatment of ischemic stroke and their positive impact on neurological recovery following injury. The therapeutic modalities involving NSCs encompass the activation of eNSCs as well as exogenous NSCs implantation. Despite numerous preclinical studies showcasing the therapeutic benefits of exogenous NSCs transplantation for neurological disorders, these effects have been consistently observed (Zhu et al., 2005, 2006; Tang et al., 2013; Jiang et al., 2019). However, the transplantation efficiency and cell survival rate of exogenous NSCs *in vivo* is low (less than 5%) (Sakata et al., 2012). Furthermore, the number of implanted exogenous NSCs that differentiate into glial cells is much higher than the number of neurons (Péron and Berninger, 2015). Exogenous NSCs implantation is not widely accepted due to concerns regarding safety, immunological response, and ethical implications. In contrast, eNSCs possess significant advantages in these areas. Firstly, eNSCs are homologous stem cells, thereby mitigating issues related to immune rejection and inflammation. Secondly, under the influence of chemokines within the body, eNSCs migrate towards damaged tissue sites and differentiate into functional neurons for repair purposes. Thirdly, on one hand, eNSCs do not pose potential risks of tumorigenicity; on the other hand, they circumvent negative effects associated with *in vitro* culture and transplantation while ensuring sustainable generation and proliferation. The experimental results demonstrated a significant decrease in the survival rate and proliferation ability of NSCs following OGD treatment. However, subsequent administration of an appropriate concentration of CART treatment led to a remarkable improvement in both the survival rate and proliferation ability of NSCs. Therefore, based on these aforementioned experimental findings, it can be concluded that CART at an optimal concentration exerts neuroprotective effects by inhibiting apoptosis in OGD-treated NSCs while promoting their proliferation. Notably, CART exerted substantial influence on the apoptosis and proliferation processes in OGD-treated NSCs; however, the specific regulatory mechanism underlying CART’s effects on OGD-treated NSCs remains unreported.

**Figure 6:**
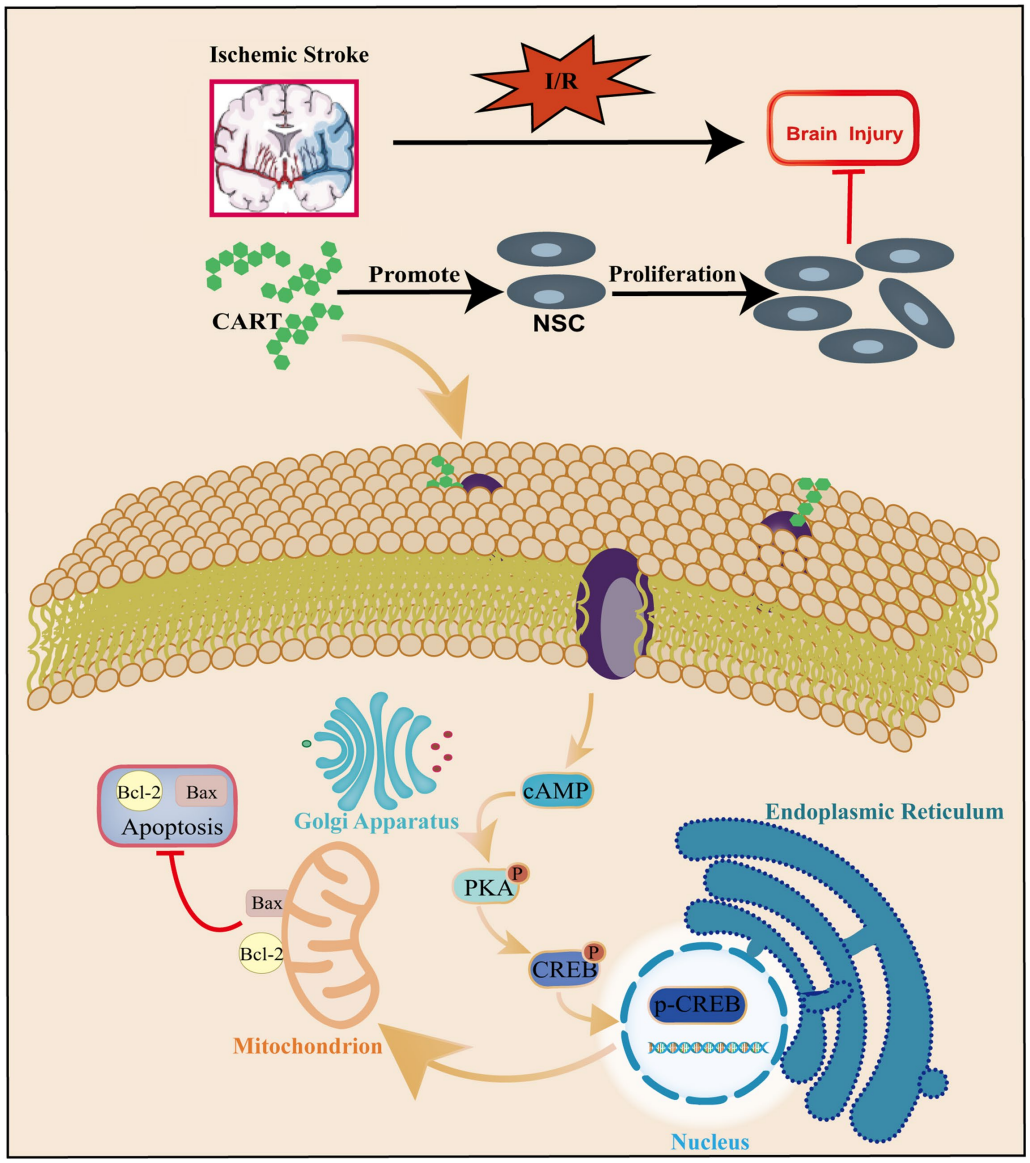
Schematic representation of the regulatory role of CART in apoptosis and proliferation of NSCs during ischemia–reperfusion.

CREB is indispensable for the growth, proliferation, differentiation, and survival of all cells. In the brain, CREB and the cAMP responsive element-mediated signaling system play crucial roles in memory formation, learning processes, synaptic transmission, neuronal viability and differentiation, as well as axon elongation (Kawashima et al., 2009; Caracciolo et al., 2018). The research has reported that CART can induce rewarding behavior by phosphorylating the PKA/ERK/CREB pathway (Somalwar et al., 2018). Our further in-depth investigation revealed that CART significantly upregulated the expression of p-CREB in NSCs subjected to OGD. Additionally, the specific inhibitor KG-501 for CREB inhibited the protective effect of CART on OGD-induced NSCs. The experimental findings suggest that CART promotes the proliferation and inhibits apoptosis of OGD-induced NSCs by activating CREB, thereby exerting a protective effect (Figure 6).

## 5 Conclusion

Taken together, CART induces nerve regeneration by activating CREB and effectively prevents ischemic stroke-induced neurological deficits. These findings help us understand the role of CART in ischemic cerebral injury and suggest that CART and its targets represent therapeutic targets in ischemic cerebral injury. Through the above studies, the novel mechanism of CART regulation of neuronal regeneration in ischemic stroke will be revealed, which will provide new ideals for the development of CART as a therapeutic drug for ischemic stroke and its receptor agonists or inhibitors. The innovation of this study lies in confirming the neuroprotective function of CART on OGD NSCs *in vitro* and determining the optimal concentration for treating OGD NSCs with CART, thereby elucidating the underlying mechanism by which it inhibits apoptosis and promotes proliferation of OGD NSCs *in vitro* to exert its neuroprotective effect on ischemic stroke. However, there are still limitations in this study. The limitation lies in the lack of further verification through animal model studies to investigate the neuroprotection of CART on ischemic stroke, as well as the need for additional research to explore whether CART can enhance neuroprotection against ischemic brain injury by regulating endogenous NSCs migration after an ischemic stroke.

## Data Availability

The raw data supporting the conclusions of this article will be made available by the authors, without undue reservation.
